# Histopathological placental lesions in mild gestational hyperglycemic and diabetic women

**DOI:** 10.1186/1758-5996-3-19

**Published:** 2011-08-10

**Authors:** Marilza VC Rudge, César P Lima, Débora C Damasceno, Yuri K Sinzato, Gustavo Napoli, Cibele VC Rudge, Franciane Q Gallego, Iracema MP Calderon

**Affiliations:** 1 Department of Gynecology and Obstetrics, Botucatu Medical School, Univ Estadual Paulista_Unesp, São Paulo State, Brazil; 2 Department of Pathology, Federal School of Medical Sciences of Porto Alegre, Brazil

**Keywords:** gestational diabetes, mild hyperglycemia, placental histopathology

## Abstract

**Objective:**

To investigate and compare the incidence of histopathological placental lesions in mild gestational hyperglycemia, gestational diabetes and overt diabetes at term and preterm gestation.

**Research design and methods:**

One-hundred-and-thirty-one placental samples were collected from *Diabetes mellitus *(DM) positive screened patients. Two diagnostic tests, glycemic profile and 100 g oral glucose tolerance test (OGTT) in parallel identified 4 groups normoglycemic, mild gestational hyperglycemia (MGH), gestational DM (GDM) or overt DM (DM). Placental tissue specimens and sections from 4 groups were obtained by uniform random sampling and stained with hematoxylin-eosin.

**Results:**

Placentas from MGH group presented 17 types of histopathological change and higher rates of syncytial nodes and endarteritis. GDM placentas presented only nine types of histopathological change, high rates of dysmaturity, low rates of calcification and no syncytial nodes. Overt DM placentas showed 22 types of histopathological change, 21 of which were present in the preterm period. There were histopathological similarities between MGH and DM placentas, but the former exhibited a higher incidence of endarteritis, which has been described as a "post-mortem" phenomenon.

**Conclusion:**

Our results confirmed that the distinct placental changes associated with DM and MGH depend on gestational period during which the diabetic insult occurs. It may reasonably be inferred that subclinical maternal hyperglycemia during pregnancy, as showed in MGH group, is responsible for increased placental endarteritis, a postmortem lesion in the live fetus.

## Introduction

The placenta is a fetal tissue that separates the maternal and fetal circulations and plays a central metabolic role in pregnancy. The placenta of diabetic women has attracted much interest, primarily because it is thought that placental damage may be partially responsible for the high incidence of fetal complications in pregnancies complicated by *Diabetes mellitus *[1]. Glucose is the main nutrient that passes to the fetus by facilitated diffusion. Thus, increased amounts of glucose may reach the fetus by facilitated transport through the placenta [2]. Therefore, adaptations in cells that are in contact with maternal and fetal circulation may occur in response to this abundant glucose supply [1].

At term, the placentas of diabetic mothers show a number of variations when compared to placentas from non-diabetic mothers. Placentas of diabetic mothers tend to be heavier [3] and exhibit immaturity of villi [4]. However, the morphological and histopathological changes in *Diabetes mellitus *placentas are inconsistent and even somewhat controversial [1]. Upon reviewing the literature, no clear picture of the effects of diabetes on the placenta emerges, most likely because of the variety of confounding factors that need to be controlled, such as type of diabetes, severity of disease, modality of treatment and quality of glycemic control. An attempt has been made to find a clinical-morphological correlation not only for manifest *Diabetes mellitus*, but also for clinically latent disturbances of carbohydrate metabolism. The timing of departures from excellent glycemic control is critical in placental development and may affect diabetes-related changes in the placenta [5].

There is controversy regarding the risk of adverse outcomes associated with types of maternal glucose intolerance that are less severe than overt diabetes [6]. Rudge used two parallel diagnostic tests, 100-g OGTT and Glucose Profile (GP), to classify pregnant women. GP measures maternal plasma glucose levels every two hours, from 8:00 AM to 6:00 PM while a standard diet of 2840 calories divided into five meals is given. A fasting glucose level of 90 mg/dL and postprandial glucose of 130 mg/dL are considered normal, and a single abnormal value confirms hyperglycemia. The combination of normal 100-g OGTT with altered GP defined a new group of pregnant women who show mild gestational hyperglycemia but do not present the criteria for gestational *Diabetes mellitus *[7]. This group can still present glucose-mediated macrosomia, perinatal mortality rates and adverse perinatal outcomes similar to those with GDM [8-18]. Studies have shown that maternal postprandial glucose concentration is the most important determinant of fetal overgrowth [19,20]. These findings demonstrate the constant risk for fetal morbidity associated with increasing maternal glucose intolerance in pregnancy [18,21].

It is not known if mild degrees of hyperglycemia are associated with the same histopathological lesions as GDM or if these lesions are still present in the preterm period. An elevation in the incidence of apoptosis and Bcl-2 in the placentas of mild gestational hyperglycemia has been demonstrated [22]. Calderon et al. [23] observed higher total area of placental terminal villi as well as a number of small villi and villous vessels, suggesting a structural adaptive response to hypoxia in the placenta [1].

The diabetic environment may have profound effects on placental development and functions [24]. Recently it was proposed that these specific effects critically depend on the time period of gestation when the insult of diabetic environment acts upon the placenta [25]. Some placental alterations continue to occur despite improvements in maternal glycemic control over the last decades, thus indicating that hyperglycemia is not the only causal factor [26,27]. We hypothesized that histopathological placental lesions are similar in patients with a wide range of glucose tolerance either in preterm or in term. Thus, the aim of the present study was to investigate and compare the incidence of histopathological lesions in placentas in mild gestational hyperglycemia, gestational diabetes and overt diabetes at term and preterm gestation.

## 2. Material and methods

### 2.1 Population

The Ethics Committee for Research of Botucatu Medical School - Unesp (Brazil) approved all procedures. The women in the study also formally consented to sample collection and the study goals. The methods used were previously by Ghidini and Salafia [28]. All placentas were prospectively collected from non-anomalous, singleton, live-born infants delivered between 34 and 38 weeks over the last year. Gestational age was established by date of last menstrual period or early ultrasonographic assessment, and the placentas were classified as term or preterm (< 37 weeks). Hospital charts were reviewed to confirm that the diagnostic criteria were met in all cases. Birth weight centiles were assigned based on the standards of Lubchenko et al. [29].

Gestational diabetes was diagnosed according to the American Diabetes Association (ADA) criteria [30] for the 100 g oral glucose tolerance test (OGTT), and mild gestational hyperglycemia was diagnosed by normal OGTT and altered glycemic profile (GP) [7]. Altered GP were considered when any two values are found or exceed fasting glycemia 90 mg/dL and post-prandial 130 mg/dL. The diagnostic tests were performed between 24 and 26 weeks of gestation in all positive screened patients [30]. Women presenting pre-gestational diabetes were included in overt diabetes group [31]. The normoglycemic group included healthy mothers with normal OGTT and normal glucose profile, delivered vaginally healthy babies at term. For the achievement of normoglycemia diabetic and mild gestational hyperglycemic patients were treated by diet alone and if necessary, by diet plus insulin

Glycemic control was assessed during pregnancy. Adequate glycemic control during pregnancy was defined as a glycemic mean of 120 mg/dl or less, and inadequate control was defined as a glycemic mean higher than 120 mg/dl.

Placental tissues were taken immediately after delivery from 131 pregnancies at Botucatu University Hospital - Unesp, a tertiary care hospital in Brazil, and classified as follows: mild gestational hyperglycemic (n = 34), gestational diabetic (n = 8), overt diabetes (n = 83) and normoglycemic (n = 6). The placental pathology examinations were performed following a standard protocol, and the same pathologist, who was blinded to the clinical data, reviewed all slides.

Placental tissues samples were collected and dissected from the central part of the placental bed and prepared for histopathological analyses. The fragments were then either immediately fixed in formalin, dehydrated in a graded ethanol series and embedded in paraffin according to a standard protocol, sectioned at 5 μm and mounted on glass slides for hematoxylin-eosin staining. The overall morphology of the placenta was evaluated under a light microscope. The placental histopathological analyses were performed in a computerized image system coupled to a photomicroscope by a digital camera. From each slide, 10 fields were randomly selected.

Twenty-two histopathological changes [32] identified in a pilot study were grouped according to etiopathogeny. Lesions of circulatory pathology included cystoid degeneration, chorial edema, edema of the intima, interstitial hemorrhage, congestion and subchorial infarction. Lesions indicative of degeneration included villus edema, villus or intervillus fibrosis, calcification and focal hyaline degeneration. Lesions of proliferation included dysmaturity, Hofbauer cell hyperplasia, chorioangiosis and syncytial nodes. Lesions of inflammation included villitis and focal amnionitis. Other lesions included "phantom cells" or cell remnants, two vessels, endarteritis, duplicate membrane and cord hemorrhage. A semiquantitative lesion score of 0 -1 was assigned based on absence or presence of histopathological changes. The total of these scores was used to calculate the total score within each group of pregnant women by gestational age (term and preterm) and glycemic control (optimal and non-optimal).

The (%) placental changes index (PCI) was calculated using the following formula:

where 22 was the number of histopathological changes observed in the placentas of diabetic women in a pilot study, and n the number of placentas evaluated in each group.

Data are presented as means or percentages. The Chi-square test (χ^2^), followed by the exact Fisher test, when necessary, was used to compare histopathological changes between groups. Unpaired Students *t*-test was used to compare maternal glycemia mean values [33]. P values of < 0.05 were considered significant.

## Results

Table 1 displays the demographic and clinical characteristics of the four groups. Women were compared considering maternal age. Maternal glycemic mean increased progressively across the diagnostic groups. Mean newborn weights were similar but prematurity was more prevalent in the overt DM compared to MGH group.

**Table 1:** Demographic, obstetric and perinatal characteristics of the study population

	Pregnant groups			
Characteristics	Normoglycemia	MGH	GDM	Overt DM
Maternal age (years)	26.7^a^	31.1 ^a^	27.1 ^a^	29.0 ^a^
Maternal glycemic mean (mg/dl)	86.2 ^a^	98.9 ^b^	114.1 ^c^	122.1^d^

Newborns				
Birth weight (grams)	3250 ^a^	3194 ^a^	3490 ^a^	3180 ^a^
Term	6 (100.0) ^a^	28 (82.3) ^a^	8 (100.0) ^a^	28 (33.7) ^a^
Preterm	0	6 (17.7) ^a^	0	55 (66.3) ^b^
LGA	1 (16.7)	4 (11.8)	2 (25.0)	23 (27.7)
AGA	5 (83.3)	28 (82.4)	6 (75.0)	55 (66.3)
SGA	0	2 (5.9)	0	5 (6.0)

Total number	6	34	8	83

Data presented as mean or number (%)

LGA: Large for Gestational Age; AGA: Adequate for Gestational Age; SGA: Small for Gestational Age

Values followed by different letters present significant statistically difference (p < 0.05)

The placental histological changes grouped according to etiopathogeny showed no significant differences (Table 2). All 22 histopathological changes were present in the placentas of the overt DM group, 21 of which were already observed in preterm period. Some histopathological changes were detected only in the placentas of GDM women: chorial edema, intimal edema, Hofbauer cell hyperplasia and villitis. The placentas of the GDM group presented only nine histopathological changes and were characterized by absence of syncytial nodes, dysmaturity and less calcification than the normoglycemic group. The placentas of the MGH group showed changes not detected in the placentas of normoglycemic group but present in diabetic women: cystoid degeneration, dysmaturity, phantom cells, two vessels in the umbilical cord, and endarteritis. The MGH group presented 17 changes, 13 of which were detected during the preterm and at term periods, these placentas showed syncytial nodes and increased endarteritis. Subchorial infarct and a duplicate membrane characterized the normoglycemic placenta.

**Table 2:** Number and percentage (%) of placental histopathological changes in women presenting normoglycemia, mild gestational hyperglycemia, gestational diabetes mellitus and overt diabetes mellitus

	Pregnant groups			
Histopathological changes	Normoglycemia	MGH	GDM	Overt DM
Circulatory				
Cystoid degeneration	0	1 (2.9)	1 (12.5)	3 (3.6)
Chorial edema	0	0	0	2 (2.4)
Intima edema	0	0	0	4 (4.8)
Interstitial hemorrhage	1 (16.7)	2 (5.9)	0	3 (4.8)
Congestion	2 (33.3)	17 (50.0)	3 (37.5)	52 (62.6)
Subchorial infarct *	2 (33.3) ^a^	3 (8.8) ^b^	0	3 (3.6) ^b^
Degenerative				
Villus edema	1 (16.7)	7 (20.6)	3 (37.5)	18 (21.7)
Villus fibrosis	1 (16.7)	8 (23.5)	0	11 (13.2)
Intervillus fibrosis	3 (50.0)	28 (82.4)	6 (75.0)	65 (78.3)
Calcification *	4 (66.7) ^a^	15 (44.1) ^a^	1 (12.5) ^b^	42 (50.6) ^a^
Focal hyaline degeneration	4 (66.7)	28 (82.4)	6 (75.0)	71 (85.5)
Proliferative				
Dysmaturity	0	5 (14.7)	3 (37.5)	8 (9.6)
Hofbauer hyperplasia	0	0	0	1 (1.2)
Chorioangiosis	1 (16.7)	4 (11.8)	0	7 (8.4)
Syncytial nodes	1 (16.7) ^a^	11 (32.4) ^b^	0	8 (9.6) ^a^
Inflammatory				
Villitis	0	0	0	2 (2.4)
Focal amnionitis	1 (16.7)	4 (11.8)	1 (12.5)	7 (8.4)
Others				
Phantom cells	0	2 (5.9)	0	1 (1.2)
Two vessels	0	1 (2.9)	0	3 (3.6)
Endarteritis	0	8 (23.5) ^a^	1 (12.5) ^b^	7 (8.4) ^b^
Duplicate membrane	2 (33.3) ^a^	0	0	1 (1.2) ^b^
Cord hemorrhage	3 (50.0)	5 (14.7)	0	14 (18.1)

Total number	6	34	8	83

Values followed by different letters present significant statistically difference (p < 0.05)

There were no significant differences in placental changes index (PCI) among the four groups within categories of gestational age and glycemic control (Table 3).

**Table 3:** Index of placental changes (IPC) in women presenting normoglycemia, mild gestational hyperglycemia, gestational diabetes mellitus and overt diabetes mellitus according to gestational age (term and preterm) and to glycemic control (adequate and inadequate)

	Pregnant groups			
	
IPC	Normoglycemia	MGH	GDM	Overt DM
Term*	19.7	19.0	14.2	20.1
Preterm*	-	24.2	-	17.3
Adequate glycemic control*	-	19.9	14.2	18.9
Inadequate glycemic control*	-	20.4	-	16.6

*p > 0.05 - non significant.

## Discussion

Our results show that placentas from MGH mothers presented more syncytial nodes and endarteritis and exhibited 17 of 22 histopathological changes. Recent studies suggest that endarteritis is a "post-mortem" lesion [34,35]. According to these authors, this lesion is not inflammatory and frequently results from thrombosis of proximal vessels. This is a microangiopathy resembling the glomerulopathy of hemolytic-uremic syndrome either in etiology or in pathology. It does not represent a specific entity but is a typical "post mortem" phenomenon [34]. Therefore, the higher endarteritis incidence in the MGH placentas may explain the high perinatal mortality rate detected in this population [7] and reinforce the new large correlation between progressive and continuous levels of maternal glycemia and adverse maternal and perinatal outcomes [36]. It is also possible that the higher incidence of endarteritis may have been responsible for the 17.7% of premature deliveries in these pregnancies. More studies are necessary to confirm this observation.

The glycemic index of food probably represents the physiological basis for the hyperglycemic levels detected in these patients. A mixed nutrient meal elicit a more robust insulin response and may be more physiologic measure of maternal hyperglycemia and subsequent macrossomia [37]. Mild hyperglycemic exchanges throughout the placenta are probably an answer to low intrauterine hypoxia and may be responsible for the alterations associated with hypervascularization of the terminal villous that were present in this organ [23]. Recently the Hapo Study (2008) identified a significant correlation between all three maternal OGTT values bellow those meeting of the exclusion criteria from diabetes study, and absolute and relative birth weight and protocol-driven neonatal c-peptide and glucose values. There is a quantitative relationship between levels of maternal glycemia and adverse maternal and perinatal outcomes and it was demonstrated to be progressive and continuous. It may reasonably be inferred that placental alterations, specifically endarteritis as a post mortem lesion and the presence of 17 from 22 histopathological changes in the MGH group, with a subclinical maternal hyperglycemia during pregnancy, which predisposes to macrosomia and late fetal death. Maternal hyperinsulinemia in MGH that persists after delivery [38] is a possible candidate mechanism to explain the high endarteritis occurrence in MGH placenta since if it persists after delivery and it could be present early in the next gestation. More studies are necessary to confirm this hypothesis. Evers et al. [39] were surprised to find that histological abnormalities of LGA-control placentas were almost comparable to those of diabetic mothers. One possible explanation is that the control women had MGH, which could only be disproven with the repeated measurement of normal glucose levels during pregnancy. These authors did not submit their patients to glucose profile, and they may be similar to the MGH group in our study [7]. The increased maternal BMI in control group this study [39] is also similar to the MGH group [38]. Despite the improvement in maternal glycemic control in GDM mothers, 25% of their newborns were classified as LGA, and all of them were at term. This population exhibited rates of LGA equivalent to those observed by Rudge et al. [7] but perinatal mortality similar to the diabetic patients treated with insulin. The placentas of GDM patients were characterized by a high incidence of dysmaturity, low indices of calcification, and absence of syncytial nodes, despite all newborns being born at term. These changes were not statistically significant but suggest delayed placental maturation. The decreased incidence of calcification might explain the delay in placental senescence observed by ultrasound in gestational diabetic patients [40].

The overt DM mothers had a higher glycemic mean during pregnancy, 27.7% prevalence of LGA and the highest incidence of prematurity among the evaluated groups. The placentas of these patients presented all 22 histopathological changes, 21 of which were already present at preterm period. The early occurrence of these histopathological changes may be a cause of the higher perinatal mortality of diabetic mothers. However, paired studies are necessary to test this hypothesis due to the different indications for optimum timing of delivery, including worsening of maternal condition, reduced insulin necessity, fetal hypoxia and fetal pulmonary maturity. The results found in the overt diabetic group confirmed the Desoye et al.' proposition [25], where specific effects depends on the time of gestation when the insult of diabetic environment acts upon the placenta. Studies of synchronized placental changes with different indications for elective preterm delivery may help to explain the non-significant correlation between clinical aspects and placental histopathology in diabetes.

In conclusion, our results confirmed that the distinct placental changes associated with DM and MGH depend on gestational period when the diabetic insult occurs, and thus on the type of diabetes [24]. It may reasonably be inferred that subclinical maternal hyperglycemia during pregnancy, as showed in MGH group is responsible not only for adverse perinatal outcome [7,36] but also for increased placental endarteritis, a postmortem lesion in the live fetus.

## Abbreviations and units

MGH: Mild Gestational Hyperglycemia; GDM: Gestational Diabetes Mellitus; OGTT: Oral Glucose Tolerance Test; PCI: Placental Changes Index; BMI: Body Mass Index; LGA: Large for Gestational Age; AGA: Adequate for Gestational Age; SGA: Small for Gestational Age; g: grams; mg/dl: milligram/deciliter.

## Competing interests

The authors declare that they have no competing interests.

## Authors' contributions

CPL and GN participated in the acquisition, analysis and interpretation of data and helped to draft the manuscript. DCD, MVCR, CVCR, FQG, YKS and IMPC participated in its design, analysis and interpretation of data and helped to draft the manuscript. All authors read and approved the final manuscript.
